# Spotted fever *Rickettsia* species in *Hyalomma* and *Ixodes* ticks infesting migratory birds in the European Mediterranean area

**DOI:** 10.1186/1756-3305-7-318

**Published:** 2014-07-10

**Authors:** Katarina Wallménius, Christos Barboutis, Thord Fransson, Thomas GT Jaenson, Per-Eric Lindgren, Fredrik Nyström, Björn Olsen, Erik Salaneck, Kenneth Nilsson

**Affiliations:** 1 Department of Medical Sciences, Section of Clinical Microbiology, Uppsala University, Uppsala, Sweden; 2 Antikythira Bird Observatory, Hellenic Ornithological Society, Athens and Natural History Museum of Crete, University of Crete, Heraklion, Greece; 3 Department of Environmental Research and Monitoring, Swedish Museum of Natural History, Stockholm, Sweden; 4 Medical Entomology Unit, Department of Organismal Biology, Uppsala University, Uppsala, Sweden; 5 Division of Medical Microbiology, Department of Clinical and Experimental Medicine, Linköping University, Linköping, Sweden; 6 Department of Medical Sciences, Section of Infectious Diseases, Uppsala University, Uppsala, Sweden; 7 Department of Medical Sciences, Clinical Microbiology, Uppsala University, SE-751 85 Uppsala, Sweden

**Keywords:** Migratory birds, Spotted fever *Rickettsia*, *Rickettsia aeschlimannii*, *Rickettsia africae*, Transmission, Tick, *Hyalomma marginatum*, *Hyalomma rufipes*, *Ixodes frontalis*

## Abstract

**Background:**

A few billion birds migrate annually between their breeding grounds in Europe and their wintering grounds in Africa. Many bird species are tick-infested, and as a result of their innate migratory behavior, they contribute significantly to the geographic distribution of pathogens, including spotted fever rickettsiae. The aim of the present study was to characterize, in samples from two consecutive years, the potential role of migrant birds captured in Europe as disseminators of *Rickettsia*-infected ticks.

**Methods:**

Ticks were collected from a total of 14,789 birds during their seasonal migration northwards in spring 2009 and 2010 at bird observatories on two Mediterranean islands: Capri and Antikythira. All ticks were subjected to RNA extraction followed by cDNA synthesis and individually assayed with a real-time PCR targeting the citrate synthase (*gltA*) gene. For species identification of *Rickettsia*, multiple genes were sequenced.

**Results:**

Three hundred and ninety-eight (2.7%) of all captured birds were tick-infested; some birds carried more than one tick. A total number of 734 ticks were analysed of which 353 ± 1 (48%) were *Rickettsia*-positive; 96% were infected with *Rickettsia aeschlimannii* and 4% with *Rickettsia africae* or unidentified *Rickettsia* species. The predominant tick taxon, *Hyalomma marginatum* sensu lato constituted 90% (*n* = 658) of the ticks collected. The remaining ticks were *Ixodes frontalis, Amblyomma* sp., *Haemaphysalis* sp., *Rhipicephalus* sp. and unidentified ixodids. Most ticks were nymphs (66%) followed by larvae (27%) and adult female ticks (0.5%). The majority (65%) of ticks was engorged and nearly all ticks contained visible blood.

**Conclusions:**

Migratory birds appear to have a great impact on the dissemination of *Rickettsia*-infected ticks, some of which may originate from distant locations. The potential ecological, medical and veterinary implications of such *Rickettsia* infections need further examination.

## Background

There are 26 validated *Rickettsia* species classified into (a) the spotted fever group (SFG) rickettsiae, (b) the typhus group rickettsiae (*Rickettsia prowazekii, Rickettsia typhi*), (c) the *Rickettsia bellii* group, and (d) the *Rickettsia canadensis* group [1-3]. *Rickettsia* species are obligate intracellular bacteria transmitted by various arthropod taxa, mainly ticks, but also fleas, lice and mites can act as vectors and reservoirs. Members of the SFG are widely distributed and several “new” species of *Rickettsia* have been described during the last few decades [1]. Seventeen of the 22 SFG-validated species and subspecies in the genus *Rickettsia* are recognized as agents of human disease of which 9 are reported in Europe [1]. Migratory passerine birds are known to be involved in the short- and long-distance spread of parasites, microorganisms and viruses of potential veterinary or medical importance. Except for SFG rickettsiae, other microbial agents including *Borrelia burgdorferi*, *Anaplasma phagocytophilum*, the tick-borne encephalitis (TBE) and the Crimean-Congo hemorrhagic fever (CCHF) viruses are known to be vectored by ticks, which sometimes infest migratory birds [4-11]. The competence of vertebrates, including birds, to function as *Rickettsia* reservoirs capable of transmitting and infecting ticks with rickettsiae is not yet completely understood [3].

*Ixodes* and *Hyalomma* are the tick genera most frequently recorded on wild birds [12]. In its adult stage, *Hyalomma marginatum* generally feeds on large mammals, while the larvae and nymphs usually prefer small mammals and ground-frequenting birds [13]. *Ixodes frontalis* is a three-host tick; which implies that a newly hatched female tick larva of this species needs to ingest blood from three different hosts before she is able to oviposit and thus to complete the life cycle. In contrast to *I. frontalis,* ticks of the *H. marginatum* species complex are two-host ticks. This signifies that the engorged *Hyalomma* larva remains on its host on which it moults to become a nymph and to take another blood meal. Therefore, a *Hyalomma* tick, which attaches as an unfed larva to a migrating bird can remain up to four weeks on the same bird. Such ticks may be transported long distances, e.g. from sub-Saharan Africa to northern Europe.

Typically, a rickettsial SFG infection presents with flulike symptoms in humans, but more severe conditions such as meningitis, perimyocarditis, facial palsy and sudden deafness may occur [14-17].

Two of the most common tick-borne spotted fever *Rickettsia* species on the African continent are *R. africae* and *R. aeschlimannii. R. africae* causes African tick bite fever. In southern Africa the bont tick (*Amblyomma hebraeum*) is the main vector and reservoir of *R. africae* in contrast to West, Central, and East Africa, where the tropical bont tick *Amblyomma variegatum* is widely distributed and serves as the main vector. *A. variegatum* has also been inadvertently introduced to several Caribbean islands where it transmits *R. africae*. [18]. *R. aeschlimannii* was first discovered in *H. marginatum* in Morocco in 1997 and has since then been found to be widespread across the continent [19]. *R aeschlimannii* has recently also been detected in countries north and north-east of the African continent. For example, in Georgia, *R. aeschlimannii* was detected in *Haemaphysalis sulcata* and in Turkey, *H. marginatum, Hyalomma aegyptium* and *Rhipicephalus bursa,* which had been removed from humans, were found to be infected with *R. aeschlimannii*[20,21]. Sometimes, an inoculation eschar develops after the infective tick bite, fever, and a generalized maculopapular rash have been recorded in patients with *R. aeschlimannii*[1,14].

Nine taxa of SFG rickettsiae are considered to be emerging pathogens of humans in Europe; *Rickettsia conorii, R. aeschlimannii, Rickettsia slovaca, Rickettsia sibirica, Rickettsia sibirica* subspecies *mongolotimoniae, Rickettsia felis, Rickettsia monacensis, Rickettsia helvetica* and *Rickettsia massiliae*[1,22].

At two Mediterranean bird observatories we collected ticks from migratory birds during two consecutive spring seasons. This study aimed to investigate if ticks, which infest migratory passerine birds in Africa are infected with *Rickettsia* species when they arrive on their avian hosts in southern Europe. This study is part of a larger investigation in which we examine the same ticks for other vector-borne human pathogens including *B. burgdorferi* s.l*.*, *Bartonella* bacteria and certain arboviruses [7,23].

## Methods

### Tick collection and bird capture

Ticks were collected from 14,789 migratory birds caught in mist nets at bird observatory stations on two Mediterranean islands: Capri (Italy; 40°33’N, 14°15’E) and Antikythira (Greece; 35°51’N, 23°18’E). The collections were performed during the birds’ northern migration in spring 2009 (March 22 to May 18) and in spring 2010 (March 20 to May 19). All birds were identified to species and examined for ticks. All removed ticks were photographed using a DinoLite Long 90x (AM4013TL) USB-microscope (AnMo Electronics Corp., Taiwan) and then placed in individual tubes containing RNAlater buffer (Qiagen GmbH, Hilden, Germany), frozen at −20˚C for subsequent freezing at −70˚C until further analysis. The identification of the stage and species of the ticks collected was based on analysis of the morphology of each tick´s dorsal and ventral sides as shown on the photographs. In some cases the tick species or stage was not possible to identify due to missing photo or poor technical quality. To confirm the species diagnosis based on tick morphology, 10 ticks chosen from the whole tick collection were subjected to a PCR-based gene sequence analysis [23]. As described previously we used the mitochondrial 12SrDNA gene as the target [23]. The results were compared with the gene sequences of *Hyalomma* species available in GenBank.

### RNA extraction, cDNA synthesis and real-time PCR

Since the survey was part of a larger study in which both viruses and bacteria were examined, both RNA and DNA were isolated from the tick samples. After homogenization of the ticks using a QIAGEN TissueLyzer (Qiagen GmbH, Hilden, Germany), RNA extraction was performed in a QIAGEN M48 BioRobot using the MagAttract® RNA Tissue Mini 48kit. For cDNA synthesis, Illustra™ Ready-to-GO RT-PCR beads kit (GE Healthcare, UK) and random hexamer primers were used [20].

All tick cDNA samples were individually assayed using a real-time PCR targeting the citrate synthase (*gltA*) gene of *Rickettsia* spp. [24]. The reactions were run in a Rotor-Gene 3000 (Qiagen, Sydney, Australia) using LightCycler® TaqMan® Master (Roche Diagnostics, Mannheim, Germany). Two to five μl cDNA was used as a template in each reaction, together with 0.25 μl LC Uracil-DNA glycosylase (UNG) (Roche Diagnostics, Mannheim, Germany) to minimize the risk of contamination. In each amplification trial a negative control, sterile water and a positive standard plasmid constructed by cloning the PCR product into a PCR 4-TOPO vector (TOPO® TA Cloning® kit for Sequencing, Invitrogen, Carlsbad, CA, USA) and containing the cloned 74 bp fragment of the *gltA* gene were included in 10-fold serial dilutions.

### Identification and genotyping of *Rickettsia* species

All samples that were positive in real-time PCR were further amplified for analysis of a fragment of the gene coding for the outer membrane protein B, *ompB*, as previously described [25]. For samples that were negative in gel electrophoresis (1% agarose) 5 μl of the PCR product from the first round were used for nesting the *ompB* PCR product yielding a 267 bp *ompB* fragment [25]. When species identification was unclear, additional PCR assays representing the 17 kDa gene and *ompA* gene were used as previously described, except for some minor changes [26-28]. The thermal cycle amplifying a fragment of the 17 kDa gene, was moderated to initial heating at 94ºC for 3 min followed by 40 repeated cycles (94°C 30s, 55°C 1 min, 72°C 1 min). The PCR assay targeting the *ompA* gene, was moderated with an initial denaturation step at 95ºC for 3 min, amplification cycle of 45 repeats with denaturation (95ºC 20s), annealing (46ºC 60s) and elongation (63ºC 60s) followed by final elongation at 72ºC for 7 min (Table 1). For analysis of the samples collected during 2010, a semi-nested PCR assay targeting the *gltA* gene was used as well [26,29]. In all conventional PCR assays, *Taq* PCR Core Kit with Q-solution was used according to the manufacturer’s instructions (Qiagen GmbH, Hilden, Germany). Negative and positive controls were included in each PCR run. Sterile water was used as the negative control. As positive controls (i) extracted DNA of *R. helvetica* isolated from a domestic *I. ricinus* tick and (ii) purified DNA of *R. conorii* were used (AmpliRun® RICKETTSIA CONORII DNA CONTROL,Vircell) [30].

**Table 1:** Details of primers, probe, product sizes and gene positions in PCR assays and sequencing of rickettsial genes

**Gene**	**Primers and probe**	**Nucleotide sequence (5’ to 3’)**	**Product size (bp)**
*gltA*	CS-F	TCG CAA ATG TTC ACG GTA CT	74
	CS-R	TCG TGC ATT TCT TTC CAT TGT G	
	CS-P	6-FAM-TGC AAT AGC AAG AAC CGT	Probe
		AGG CTG GAT G--BBQ-1	
*ompB*	Rc.rompB.4362p	GTC AGC GTT ACT TCT TCG ATG C	475
	Rc.rompB.4,836n	CCG TAC TCC ATC TTA GCA TCA G	
	Rc.rompB.4,496p	CCA ATG GCA GGA CTT AGC TAC T	267
	Rc.rompB.4,762n	AGG CTG GCT GAT ACA CGG AGT AA	
17 kDa	Rr17kDa.61p	GCT CTT GCA ACT TCT ATG TT	434
	Rr17kDa.492n	CAT TGT TCG TCA GGT TGG CG	
*ompA*	Rr 190.70 F	ATG GCG AAT ATT TCT CCA AAA	632
	Rr 190.701R	GTT CCG TTA ATG GCA GCA TCT	
*gltA*	RH314	AAA CAG GTT GCT CAT CAT TC	857
	CSF-R	AAG TAC CGT GAA CAT TTG CGA	
	CS-Ric-R	CAG TGA ACA TTT GCG ACG GTA	852
	CS535d	GCA ATG TCT TAT AAA TAT TC	Sequencing
			Primer

PCR reactions were performed in a GeneAmp® PCR System 9700 (Applied Biosystems, Foster City, CA), and expected PCR products were confirmed using gel electrophoresis (1% agarose) stained with 2% ethidium bromide or GelRed™ (Biotium Inc). All PCR products considered for sequencing were cleaned using Exonuclease I and FastAP^TM^ Thermosensitive Alkaline Phosphatase (Fermentas GmbH). Direct cycle sequencing of PCR products from 2009 year’s samples was performed using BigDye® Terminator v 3.1 Cycle Sequencing Kit in an ABI 3130 instrument (Applied Biosystems). PCR products from 2010 year’s samples were sent for sequencing analysis at Macrogen Inc. (Macrogen Europe, Amsterdam, Netherlands). Sequence alignments and analysis were performed using DNA Baser version 2.80.0 (HeracleSoftware, Lilienthal, Germany) and BioEdit Sequence Alignment Editor Version 7.0.5.3 (Ibis Therapeutics, Carlsbad, CA). For species identification, similarities and differences between sequences were examined using the Basic Local Alignment Search Tool (BLAST).

### Ethics Statement

Trapping of birds was approved on Capri by the Board of the Italian National Ringing Centre and on Antikythira by “the Ministry of Agriculture” and the Greek Ringing Centre. Ethical approval for sampling ticks from birds was obtained from the Board of the European Animal Research Ethics Committee.

## Results

### Birds and tick infestation

#### Study 2009

The first spring season’s trapping on Antikythira resulted in 2,522 migratory birds, representing 55 different species that were examined for tick infestation. In a corresponding manner, 4,913 migratory birds on Capri were also checked, representing 49 species of birds. Overall, 376 ticks were collected from a total of 195 birds, of which 137 ticks were collected from 65 birds on Antikythira and 239 ticks from 130 birds on Capri.

#### Study 2010

During spring on Antikythira, 3,332 birds, representing 54 different species, were captured and checked for tick infestation, resulting in a total of 200 ticks from 103 birds. Correspondingly, on Capri 4,022 birds, representing 49 species were examined, resulting in a total of 158 ticks from 100 birds.

#### Birds

When combining data from both bird observatories, both years, 79 species of birds were caught and examined for ticks, 27 species where found to be tick infested. The number of birds that belonged to any of the 27 species was 13,322 individuals of the total 14,789 caught birds. The species with the highest infestation of ticks per individual bird was the Woodchat Shrike (*Lanius senator*), which carried 53 ticks on 122 birds. The Whinchat (*Saxicola rubetra*) was one of the most common birds from which a total of 1,472 individual ticks was caught. However, this species was less burdened by ticks per individual bird but was also the species that contributed the largest number of ticks (135). Almost as many ticks, 123, were brought in by the 1,244 Common Whitethroats (*Sylvia communis*) (Table 2).

**Table 2:** Birds infested by Rickettsia spp.-infected ticks

**Bird species**		**No. *Rickettsia *** **spp. infected ticks (total no. of ticks)/No. birds carrying ticks infected with *Rickettsia *** **spp. (No. birds carrying ticks)**									
		**2009**				**2010**				**Total number included in study**	
**Latin name**	**Common name**	**Antikythira**		**Capri**		**Antikythira**		**Capri**			
		**Ticks**	**Birds**	**Ticks**	**Birds**	**Ticks**	**Birds**	**Ticks**	**Birds**	**Ticks**	**Birds**
*Acrocephalus arundinaceus*	Great Reed Warbler					8^1^ (11)	2 (3)			11	197
*Acrocephalus schoenobaenus*	Sedge Warbler	10^2^ (16)	5 (6)			15 (23)	4 (7)			39	459
*Acrocephalus scirpaceus*	Reed Warbler	3 (4)	1 (1)			0 (2)	0 (1)			6	24
*Anthus trivialis*	Tree Pipit	1 (4)	1 (4)	3 (7)	2^3^ (3)	2 (5)	2 (5)			16	406
*Caprimulgus europaeus*	Eurasian Nightjar					2 (2)	1 (1)			2	25
*Carduelis chloris*	European Greenfinch					1^4^ (4)	1 (1)			4	21
*Carduelis spinus*	Siskin					1^5^ (1)	1 (1)			1	3
*Erithacus rubecula*	European Robin	2 (4)	1 (1)			0 (12)	0 (6)			16	127
*Fidecula albicollis*	Collard Flycatcher	3 (3)	1 (1)			0 (3)	0 (2)			6	174
Fidecula hypoleuca*	European Pied Flycatcher	2 (7)	2 (7)	20 (44)	15 (31)	0 (8)	0 (4)	5^6^ (31)	4 (22)	59	2008
Hippolais icterina*	Icterine Warbler	2 (3)	1 (2*)	1 (1*)	1 (1*)	1 (1)	1 (1)	0 (2)	0 (1)	7	475
*Hippolais pallida*	Olivaceous Warbler	1 (1)	1 (1)			1 (1)	1 (1)			2	46
*Lanius senator*	Woodchat Shrike	20 (25)	4 (5)	3 (3)	2 (2)	12 ± 1 (25)	8 (11)			53	122
*Luscinia megarhynchos*	Common Nightingale	12 (15)	3 (6*)	0 (1)	0 (1)	11 (20)	4 (7)			36	319
*Motacilla flava*	Yellow Wagtail	9 (9)	1 (1)			1 (2)	1 (1)			11	9
*Muscicapa striata*	Spotted Flycatcher			2 (2)	2 (2)	1 (1)	1 (1)	3 (4)	3 (4)	7	1187
*Oenanthe oenanthe*	Wheatear					2 (2)	2 (2)	13 (17)	4 (4)	19	82
Oriolus oriolus*	Golden Oriole	0 (2)	0 (2)	4 (9)	3 (4)	3^7^ (6)	3 (5)	0 (1)	0 (1)	18	293
Phoenicurus phoenicurus*	Common Redstart	5 (10)	3 (6)	3 (10)	3 (6)	10^8^ (24)	10 (15)	1 (7)	1 (4)	51	386
*Phylloscopus orientalis*	Eastern Bonelli's Warbler					3 (7)	3 (4)			7	33
*Phylloscopus sibilatrix*	Wood Warbler	1 (5)	1 (4)	6 (13)	5 (12)	2 (5)	2 (5)	3 (10)	3 (9)	33	1238
*Phylloscopus trochilus*	Willow Warbler	1 (2)	1 (2)	1 (1)	1 (1)			0 (1)	0 (1)	4	738
Saxicola rubetra*	Whinchat	0 (2)	0 (2)	16 (73)	13 (33)	1 (3)	1 (2)	23 (58)	14 (34)	135	1472
Sylvia borin*	Garden Warbler	2^9^ (3)	2 (3)	0 (3)	0 (1)	3 (4)	3 (4)	1 (3)	1 (3)	13	2190
Sylvia communis*	Common Whitethroat	10 (16)	6 (8)	43 (70)	24 (33)	8 (13)	6 (9)	16 (24)	10 (17)	123	1244
*Turdus philomelos*	Song Thrush	4 (5)	1 (1)			7^10^ (13)	2 (2)			18	23
*Upupa epops*	Eurasian Hoopoe					2 (2)	2 (2)			2	18
Unknown species		0 (1)	0 (1)	1 (2)	1 (2)					3	3
	27 species	88 (137)	36 (65)	103 (239)	72 (130)	97 ± 1 (200)	61 (103)	65 (158)	40 (100)	353 ± 1 (734)	13 322**

Of the infected ticks 96% carried *Rickettsia aeschlimannii*, marked are those ticks who carried other rickettsia species, see Tablee 5 for more details. 1. One *Rickettsia* sp. (84748).

2. Two Rickettsia sp. (84607 and 84609) 3. One *Rickettsia* sp. (84154) from an *Ixodes frontalis* adult female 4. One probable *R. africae* 5. One *R. africae 6.* One possible *R. monacensis* (113618) from an *Ixodes frontalis* 7. One *Rickettsia* sp. (84751) 8. One *R. africae 9.* One *Rickettsia* sp. (84594) from a *Haemaphysalis* sp. nymph 10. Four *R. africae* and one probable *R. africae* *One bird with uncertain species definition. **Bird species without ticks are not included.

#### Ticks

The samples from 2009 and 2010 included a total of 751 ticks of which 734 were available for analyses. The most common stage was nymphs of which 488 individual ticks were collected. The remaining ticks were 198 larvae, 4 adults and 44 ticks of unidentified stage, the latter due to missing photos or photographs of poor technical quality. Two tick samples were for practical reasons pooled, one larva with one nymph and two nymphs with one larva. The number of ticks, their species and developmental stages are presented in Table 3. In total 398 birds were infested and relieved of their ticks (Table 2). The number of ticks per infested bird ranged from 1 to 20; 37% (*n =* 148) of the birds carried more than one tick. Of all ticks that could be evaluated (567/683), 83% of the ticks were generally fully or partially fed. Most ticks belonged to *H. marginatum* sensu lato (s.l), which constituted 90% (*n* = 658 samples) of the tick collection. Out of ten gene-sequenced ticks, nine were identified as *H. rufipes*; and one as *H. marginatum* sensu stricto, which supported the species diagnoses based on morphology [20]. Twenty-seven ticks of the genus *Ixodes* were collected at the bird observatories. Twenty-three were identified as *I. frontalis*. Eight of these *Ixodes* spp. ticks were parasitizing on European Robins (*Erithacus rubecula*). Two uninfected *Rhipicephalus* sp. nymphs and two *Haemaphysalis* sp. nymphs were also collected. One of the *Haemaphysalis* sp. nymphs (tick 84594, Tables 4 and 5) were caught on a Garden Warbler (*Sylvia borin*) at Antikythira 2009 infected with *Rickettsia* sp. and the other one, which infested a Wood Warbler *(Phylloscopus sibilatrix)* at Antikythira 2010, was infected with *R. aeschlimannii*.

**Table 3:** **Tick species and rate of infection with *Rickettsia *** **spp. per stage of development**

**Tick species**	**Ticks stage**	**No. infected with *R. aeschlimannii***	**No. infected with *R. africae***	**No. infected with *Rickettsia *** **sp.**	**No. infected ticks (total number ticks)**
***Hyalomma marginatum *** **s.l.**	Larva	73	11	7^1^	81 (189*)
	Nymph	227 ± 1**	4	9^2^	240 ± 1** (459)
***Hyalomma rufipes***	Larva			1^3^	**1 (2)**
	Nymph	7			**7 (7)**
***Hyalomma marginatum *** **s.s.**	Nymph	1			**1 (1)**
***Ixodes frontalis***	Larva				**0 (4)**
	Nymph	4		1^4^	**5 (15)**
	Adult (female)			1^5^	**1 (4)**
***Ixodes *** **spp.**	Larva				**0 (3)**
	Nymph				**0 (1)**
***Amblyomma *** **sp.**	Nymph				**0 (1)**
***Haemaphysalis *** **sp.**	Nymph	1		1^6^	**2 (2)**
***Rhipicephalus *** **sp.**	Nymph				**0 (2)**
**Unidentifiable species**	Unididentifable stage stage	5	1	9^7^	**15 (44)**
***Total***		**318 ± 1**	**6**	**29**	**353 ± 1 (734)**

*One larva was pooled with one nymph, **One pool consisted of two nymphs and one larva. 1. One *Rickettsia* sp. (84751) and six probable *R. aeschlimannii,* 2. Two *Rickettsia* sp. (84748 and 84607) and seven probable *R. aeschlimannii* 3. One *Rickettsia* sp. (84609), 4. One possible *R. monacensis* (113618), 5. One *Rickettsia* sp. (84154) 6. One *Rickettsia* sp. (84594), 7. Two probable *R. africae* and seven probable *R. aeschlimannii.*

**Table 4:** **Ticks infected with other *Rickettsia *** **spp. than *Rickettsia aeschlimannii***

**Sample location**	**Bird species**	**Tick species**	**Tick stage**	***Rickettsia *** **species**	Sample ID*
Capri 2009	Tree Pipit	*Ixodes frontalis*	Female	*Rickettsia* sp.	84154
Antikythira 2009	Garden warbler	*Haemaphysalis* sp.	Nymph	*Rickettsia* sp.	84594
	Sedge warbler	*H. marginatum* s.l.	Nymph	*Rickettsia* sp.	84607
		*Hyalomma rufipes*	Larva	*Rickettsia* sp.	84609
Capri 2010	European pied flycatcher	*Ixodes frontalis*	Nymph	Possibly *R.monacensis*	113618
Antikythira 2010	Common redstart	*H. marginatum* s.l.	Larva	*R. africae*	
	Song thrush	*H. marginatum* s.l.	Nymph	*R. africae*	
		*H. marginatum* s.l.	Nymph	*R. africae*	
		*H. marginatum* s.l.	Nymph	*R. africae*	
	Eurasian siskin	*H. marginatum* s.l.	Nymph	*R. africae*	
	Song thrush	No photo		*R. africae*	
		No photo		*R. africae* (probable)	
	European greenfinch	No photo		*R. africae* (probable)	
	Great reed warbler	*H. marginatum* s.l.	Nymph	*Rickettsia* sp.	84748
	Golden oriole	*H. marginatum* s.l.	Larva	*Rickettsia* sp.	84751
	**11 Individual birds**	**15 Individual ticks**			

*Sequence data for these samples are displayed in Table 6.

**Table 5:** Outcome of Blast hit comparison in determining the identity of amplified sequences

**Sample ID**	***ompB***	**BLAST hit**	***17 kDa***	**BLAST hit**	***gltA***	**BLAST hit**	***ompA***	**BLAST hit**
84154	219/222	Candidatus *R. amblyommii* [CP003334/JN378402]	nd		nd		nd	
		*R. helvetica* [HQ232249/HQ232247]						
		*R. amblyommii* [AF479763]						
		Uncultured Rickettsia sp. clone CsfC2 [EU407140]						
84594	424/431	Rickettsia sp. T170-B [JQ727680]	394/394	*R. raoultii*	nd		nd	
		*R. heilongjiangensis* [CP002912/AY280712]*		Rickettsia sp. RpA4				
		Rickettsia sp. IG-1 [EF219461]		Rickettsia sp. ARANHA				
				Candidatus *R. gravesii*				
84607	428/431	*R. slovaca* [CP003375/CP002428]*	392/392	Candidatus *R. antechini* [DQ372953]	nd		nd	
		*R. parkeri* [CP003341/FJ644549]*	392/394	*R. rickettsii* [KC845924/CP003311]*				
		*R. sibirica* [HM050273/AF123722]		*R. parkeri* [CP003341/EF689732]*				
		*R. conorii* [AE006914/AF123726]*		*R. philipii* [CP003308]				
		Rickettsia sp. BJ-90 [AY331393]		*R. peacockii* [CP001227/AF260571]*				
				Rickettsia sp. NOD [EU567178]				
84609	220/222	*R. slovaca* [CP003375/CP002428]*	392/392	Candidatus *R. antechini* [DQ372953]	nd		nd	
		*R. parkeri* [CP003341/FJ644549]*	392/394	*R. rickettsii* [KC845924/CP003311]*				
		*R. rickettsii* [CP003311]		*R. parkeri* [CP003341/EF689732]*				
		*R. philipii* [CP003308]		*R. philipii* [CP003308]				
		*R. sibirica* [HM050273/AF123722]		*R. peacockii* [CP001227/AF260571]*				
		*R. conorii* [AE006914/AF123726]*		Rickettsia sp. NOD [EU567178]				
		*R. mongolotimonae* [DQ097083/AF123715]						
		Rickettsia sp. BJ-90 [AY331393]						
		Uncultured Rickettsia sp. clone B3[DQ019321]						
84748	185/187	Candidatus *R. hoogstraalii* [EF629536]	392/392	Rickettsia sp. Torishima-CC1[AB242434]	734/734	*R. endosymbiont* [DQ081187]	nd	
			387/387**	Rickettsia sp. Scc31 [DQ105801]				
			386/386**	*R. endosymbiont* [DQ081185]				
			382/382**	Candidatus *R. hoogstraalii* [FJ767736]				
84751	427/431	Rickettsia sp. T170 [JQ727680]	394/394	Rickettsia sp. LON-13 [AB516961]	791/791	Rickettsia sp. Mie180 [JQ697958]	584/587	Rickettsia sp. HlR/D91 [KC888951]
		*R. heilongjiangensis* [CP002912/AY280712]*		Rickettsia sp. LON-2 [AB516960]		Rickettsia sp. LON-13 [AB516964]	583/587	Rickettsia sp. FUJ98 [AF169629]
				*R. marmionii* [AY737683]			485/488**	Rickettsia sp. LON-13 [AB516963]
				Rickettsia sp. Hf151 [AB114816]				
				Rickettsia sp. Hl550 [AB114805]				
113618	222/222	*R. monacensis* [JX625150]	nd		nd		nd	

*Two examples **The deponated sequence at NCBI GenBank is shorter than our sequence.

#### Rickettsia spp. infection of ticks

Of the 14,789 captured birds 2.7% (398/14,789) were infested with ticks; 53% (209/398) of these birds carried ticks positive for *Rickettsia* spp. The overall *Rickettsia* infection rate was 52% for nymphs and 41.5 ± 0.5% for larvae. The prevalence and distribution of *Rickettsia* species in the 353 ± 1 PCR-positive ticks, in relation to collection site and year, are summarized in Table 6. Multiple-infested birds occurred, and one individual Sedge Warbler (*Acrocephalus schenobaenus*) carried eight tick larvae (Tables 2, 3, 4 and 5), three of which were infected with *R. aeschlimannii*, three negative larvae (all fully fed) and two larvae with unidentified species of *Rickettsia* (tick 84607 and 84609, Table 5). One Woodchat Shrike (*Lanius senator),* had 19 engorged *H. marginatum* s.l. ticks, of which four nymphs were negative for *Rickettsia* DNA and 12 nymphs and three larvae were infected with *R. aeschlimannii*. A Great Reed Warbler (*Acrocephalus arundinaceus)* caught on Antikythira carried six nymphs, four of which were infected with *R. aeschlimannii,* one with an unidentified *Rickettsia* (tick 84748, Table 5), while the sixth nymph was negative for *Rickettsia* spp. Regarding the predominant tick species complex *H. marginatum* s.l.*,* 330 ± 1 of 658 ticks were infected with *Rickettsia* spp., 93% of which were identified as *R. aeschlimannii*.

**Table 6:** **Prevalence, collection site and year of *Rickettsia *** **species in PCR-positive ticks**

	**Antikythira**		**Capri**		**Total**
	**2009**	**2010**	**2009**	**2010**	
*R. aeschlimannii*	82	80 ± 1	99	57	318
Probable *R. aeschlimannii*	3	7	3	7	20
*R. africae*		6			6
Probable *R. africae*		2			2
*Rickettsia* spp.	3	1	1		5
Possible *R. monacensis*				1	1
Possible *Rickettsia* sp. LON-13		1			1
**Subtotal**	88	97 ± 1	103	65	
**Total**	**185 ± 1**		**168**		**353 ± 1**

Among 27 *Ixodes* ticks, five *I. frontalis* nymphs were infected with *Rickettsia* spp. Four of these nymphs, all collected in 2010 on Antikythira, were infected with *R. aeschlimannii* and the fifth nymph, collected from a Pied Flycatcher (*Ficedula hypoleuca)* at Capri, was probably infected with *R. monacensis* (only the nested PCR product of the *ompB* gene was amplified and sequenced; tick 113618, Table 5).

In this study only four adult ticks were collected and all were *I. frontalis* females, three of which were uninfected. The fourth adult tick was removed from a Tree Pipit (*Anthus trivialis)* at Capri and was infected with an unidentifiable *Rickettsia* species (tick 84154, Table 5).

### *Rickettsia* species composition

The prevalence of *Rickettsia* spp. in all 734 tick samples was determined by analysing all samples positive by real-time PCR. The real-time PCR results stretched from 3 to 9.0 *10^6^ copies of the *gltA* gene/μl, median 175000 copies/μl. If there were successful amplification and sequencing of any of the genes from the conventional PCRs, samples were considered positive for *Rickettsia*. When comparing the amplified sequences the primer regions were excluded.

#### R. aeschlimannii

Species identification of *Rickettsia* by sequencing the fragment of the *ompB* gene was successful in 306 cDNA samples, 300 of the obtained sequences matched *R. aeschlimannii* [GenBank: AF123705, HM050278] on 430 of 431 positions. All sequences of *R. aeschlimannii* were identical and shared a transition in one position referred to as base number 4549 compared to the deposited sequence AF123705 and also a transition on base number 4473 compared to the deposited sequence HM050278. Both transitions were located in the middle of the common sequence where readings of both primers overlap, making it more likely to be an accurate change. The variation found in the above-mentioned position 4549, compared to AF123705, does not alter the protein sequence, however, the nucleotide exchange compared to HM050278 alters the protein sequence from amino acid alanine to valine, therefore, this sequence has been annotated [GenBank: KF646134]. In case the single PCR for *ompB* did not yield any product or if there were difficulties in determining rickettsial identity, the samples were rerun with a nested PCR for *ompB* or PCR for the *ompA*, 17 kDa or *gltA* genes (Table 1). The nested *ompB* PCR yielded a 222-bp-long product (when subtracting the primer regions) for sequence analysis, and in relation to *R. aeschlimannii,* the difference compared to the closest species, *R. raoultii,* is only 2 nt. For 15 of the samples, sequencing of the nested *ompB* product was complemented with either one or both of the *gltA* and *ompA* (590 bp) sequences, where the products matched 100% with *R. aeschlimannii* [GenBank: HM050289 (*gltA*), HM050290 (*ompA*)]. For two samples, amplification of an *ompB* sequence was unsuccessful. These samples were instead amplified and sequenced for both the *gltA* and *ompA* genes, and both had a 100% sequence match to *R. aeschlimannii*. This finally resulted in 318 ± 1 ticks with confirmed *R. aeschlimannii* infection and a further 20 samples that were only successfully sequenced for the shorter nested part of the *ompB* sequence and judged as probable *R. aeschlimannii*. From samples already characterized as *R. aeschlimannii*, 114 were chosen for sequencing of the amplified 17 kDa gene product. The currently deposited sequences in GenBank representing the 17 kDa gene of *R. aeschlimannii*, of which the longest sequence [GenBank: DQ379979] is 357 bp, are all shorter than products amplified in this project. When excluding the primer region the sequences analysed was 394 bp long. However, the corresponding part of the deposited sequences all matched 100% with 67 of the 114 sequences. The remaining 47 sequences have an A in position 237, compared to deposited sequences that have a G nucleotide in position 237. Both sequence variants found have been deposited to GenBank and annotated accession numbers KF646135 and KF646136. The two bird species, the Whinchat and the Common Whitethroat that contributed with most of the ticks only carried ticks infected by *R. aeschlimannii*.

#### R. africae

Six ticks were infected with *R. africae*. Amplification and sequencing parts of the *ompB*, *ompA* and *gltA* genes yielded sequences with 100% similarity to the deposited sequences in GenBank, e.g. HQ335130. All ticks infected with *R. africae* were collected on Antikythira in spring 2010 from four different birds. Five ticks were *H. marginatum* s.l. and the sixth was not identifiable. One infected larva infested a Redstart (*Phoenicurus phoenicurus*). Four of the other findings of *R. africae* were from nymphs; three of which came from the same Song Thrush (*Turdus philomelos*), together with two nymphs negative for *Rickettsia* spp. The fourth infected nymph was one single tick infesting one of a total of three collected Eurasian Siskin (*Carduelis spinus*). The unidentified sixth tick was collected from another Song Thrush, together with two unidentifiable ticks infected with *R. aeschlimannii*, four PCR negative ticks and one unidentifiable tick that probably was infected with *R. africae*, (Table 6). A second probable finding of *R. africae* was also in an unidentifiable tick collected from a European Greenfinch (*Carduelis chloris*), together with three PCR negative ticks. The nested *ompB* sequence for *R. africae* has only one nucleotide distinction from a number of different SFG rickettsae, among others *R. rickettsii, R. slovaca*, *R. conorii* strain Indian, *R. mongolotimonae* and *R. sibirica.* This is why the two samples only successfully sequenced for the short (222 bp) part of *ompB,* with a 100% match for *R. africae* [GenBank: HQ335130] were considered as probably *R. africae*. All six samples characterized as *R. africae* were also successfully sequenced for the amplified part of the 17 kDa gene, which shared 100% (394/394 bp) similarity with *Rickettsia* sp. HymargITA12 [GenBank: AJ781419] and 99% (390/394 bp) with *R. africae* ESF-5 [GenBank: CP001612]. The 394 bases yielded an open reading frame of 131 amino acids with two of the base substitutions changing the amino acid composition of the protein from lysine to arginine on amino acid position 72 and from valine to isoleucine on position 96. None of the shorter *R. africae* 17 kDa sequences annotated in GenBank had 100% similarity with the 394-bp-long sequence found, it has been deposited to GenBank as *R. africae* [GenBank:KF616137]. 

#### Rickettsia spp.

In seven ticks, rickettsial DNA distinct from *R. aeschlimannii* and *R. africae* was detected. Two of these ticks, one newly hatched *H. marginatum* s.l. nymph and one *H. rufipes* larva (84607 and 84609, Table 5) were collected from a Sedge Warbler on Antikythira in 2009. These ticks were positive for rickettsial DNA and shared identical sequences for the 17 kDa gene. They also had identical *ompB* sequences, though one of the ticks was positive in the first PCR while the other one was only positive in the nested *ompB* PCR. The *Rickettsia* sp. matched closely to the cluster of *R. conorii, R. rickettsii*, *R. parkeri*, *R. africae* and *R. slovaca* but the correspondence was not 100% for any of the two sequences. One *I. frontalis* nymph (tick 113618) had a relatively low number of copies (978 copies/ μl) in the real-time PCR and only the nested product of the *ompB* gene was possible to amplify. Sequencing of the amplified product with subtraction of primer regions, gave a 100% match with 222 bp of *R. monacensis* [GenBank: JX625150]. One of six *H. marginatum* s.l. nymphs (tick 84748, Table 6) collected from a Great Reed Warbler trapped on Antikythira, was infected with a *Rickettsia* species, of which a 187 bp fragment of the *ompB* gene was sequenced and 185 bp matched with Candidatus *Rickettsia hoogstraalii* [GenBank: EF629536]. For the *gltA* gene 734 bp was amplified and matched 100% with *Rickettsia endosymbiont* [GenBank: DQ081187] and the 17 kDa gene was successfully sequenced for the whole fragment and the closest match was 392/392 bp with *Rickettsia* sp. Torishima-CC1 [GenBank: AB242434]. For *R. endosymbiont* and Candidatus *R. hoogstraalii,* shorter 17 kDa sequences are annotated in GenBank and the sequence from tick 84748 matched 100% with both, 386/386 [DQ081185] and 382/382 [FJ767736]. Four of the other five nymphs collected from the same bird were infected with *R. aeschlimannii*.

A Golden Oriole (*Oriolus oriolus*) captured on Antikythira in 2010, carried one *Hyalomma* larva (tick 84751) infected with a *Rickettsia* sp. Sequencing the *gltA* and 17 kDa gene amplified products that both matched 100% with *Rickettsia* sp. LON-13 [GenBank: AB516964, AB516961]. The *ompA* sequence from the same sample matched the shorter annotated LON-13 sequence on 485/488 bp [GenBank: AB516963]. For *ompB,* there are no sequences annotated in NCBI GenBank in relation to LON-13.

Blast results for all seven *Rickettsia* sequences, the small sequence differences and the importance of assessing multiple sequences before species identity is determined, are illustrated in Table 5[31].

## Discussion

**Figure 1:**
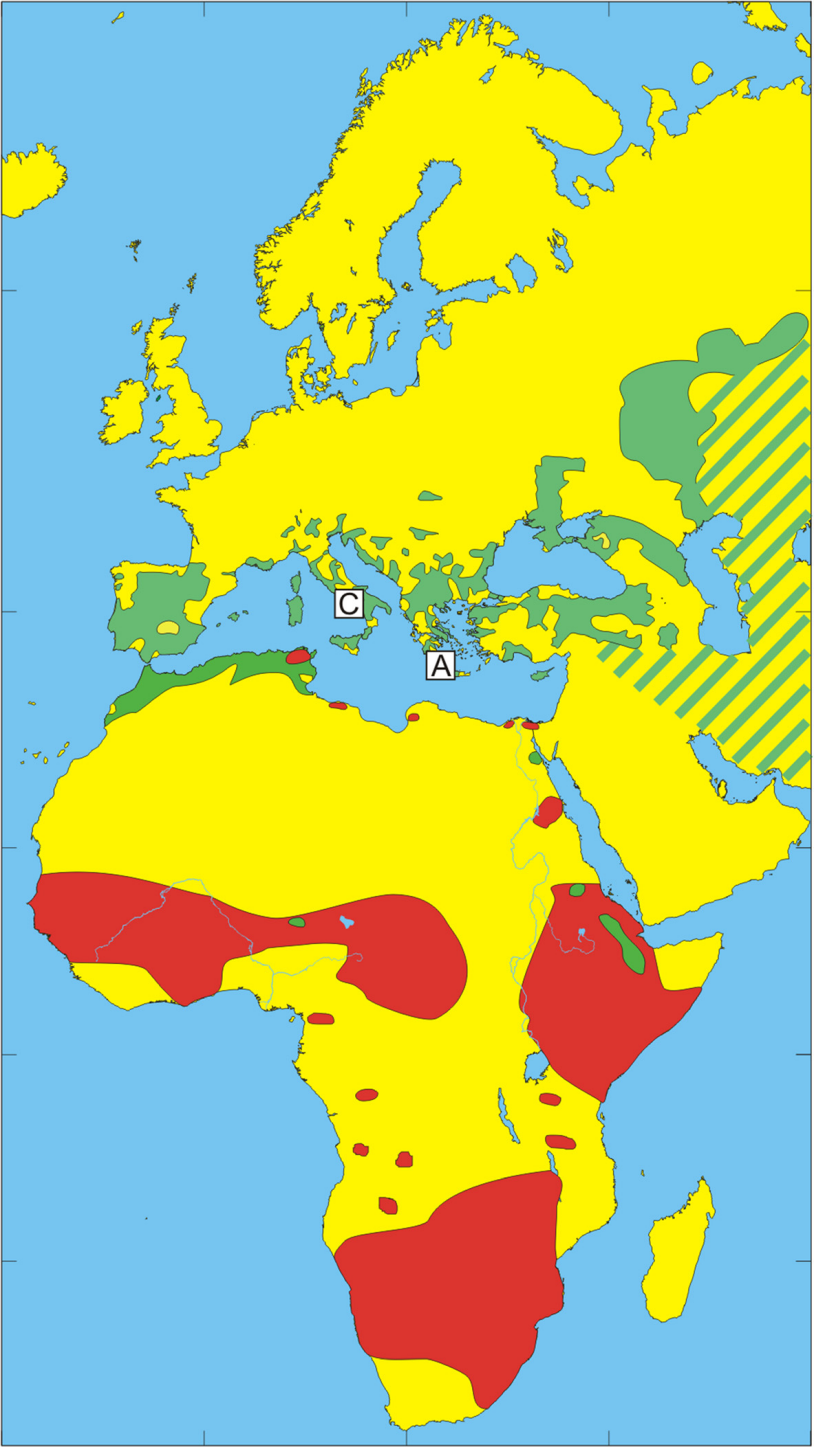
**Tick-distribution maps.** Areas from where Hyalomma marginatum (green) and Hyalomma rufipes (red) have been reported. Based on tick maps from the “European centre for disease prevention and control” and Walker et al. [34,35].

**Figure 2:**
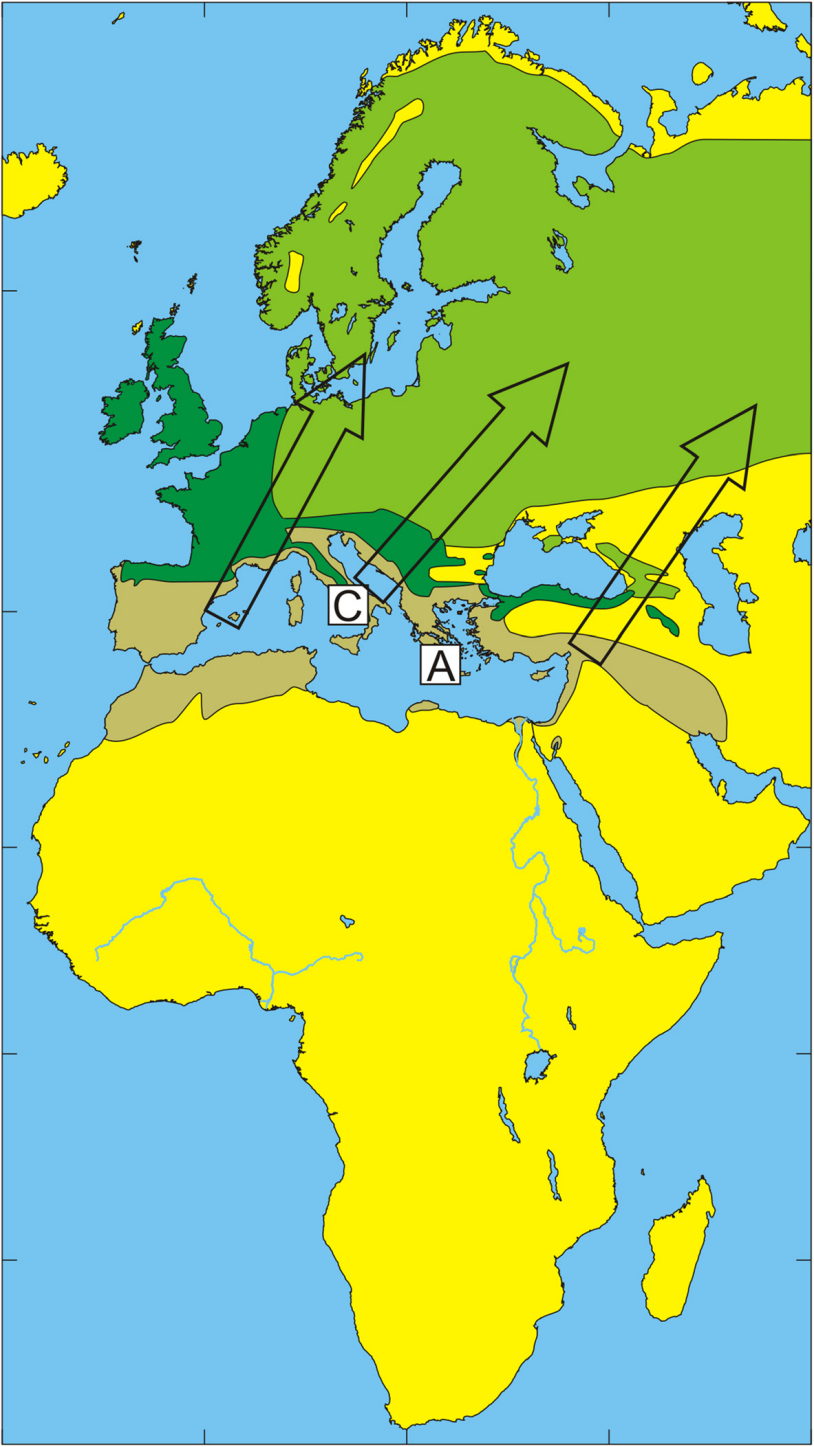
**Illustration of the wintering and breeding areas and migration directions of five bird species.** Breeding (green) and wintering (light brown) areas for some bird species included in the study, based on Cramp and Perrins [36-38]. Dark green indicates areas where birds are present all year around. Arrows show the main direction of movements from wintering areas towards breeding areas during spring migration.Song Thrush *Turdus philomelos*. The locations of Capri (C) and Antikythira (A) bird observatories in Italy and Greece, respectively, are shown by white squares.

**Figure 3:**
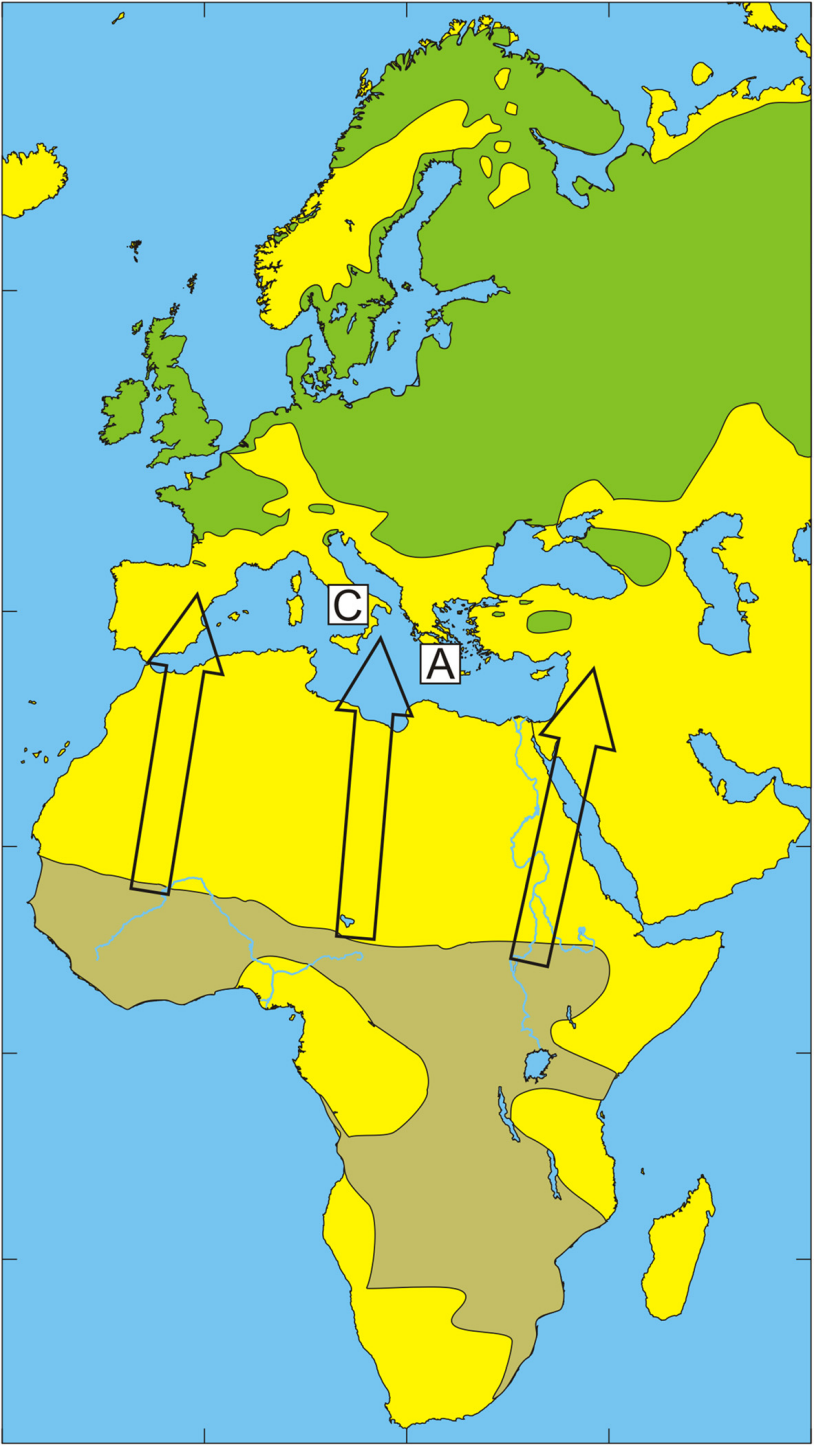
**Illustration of the wintering and breeding areas and migration directions of five bird species.** Breeding (green) and wintering (light brown) areas for some bird species included in the study, based on Cramp and Perrins [36-38]. Arrows show the main direction of movements from wintering areas towards breeding areas during spring migration.Sedge Warbler *Acrocephalus schoenobaenus.* The locations of Capri (C) and Antikythira (A) bird observatories in Italy and Greece, respectively, are shown by white squares.

**Figure 4:**
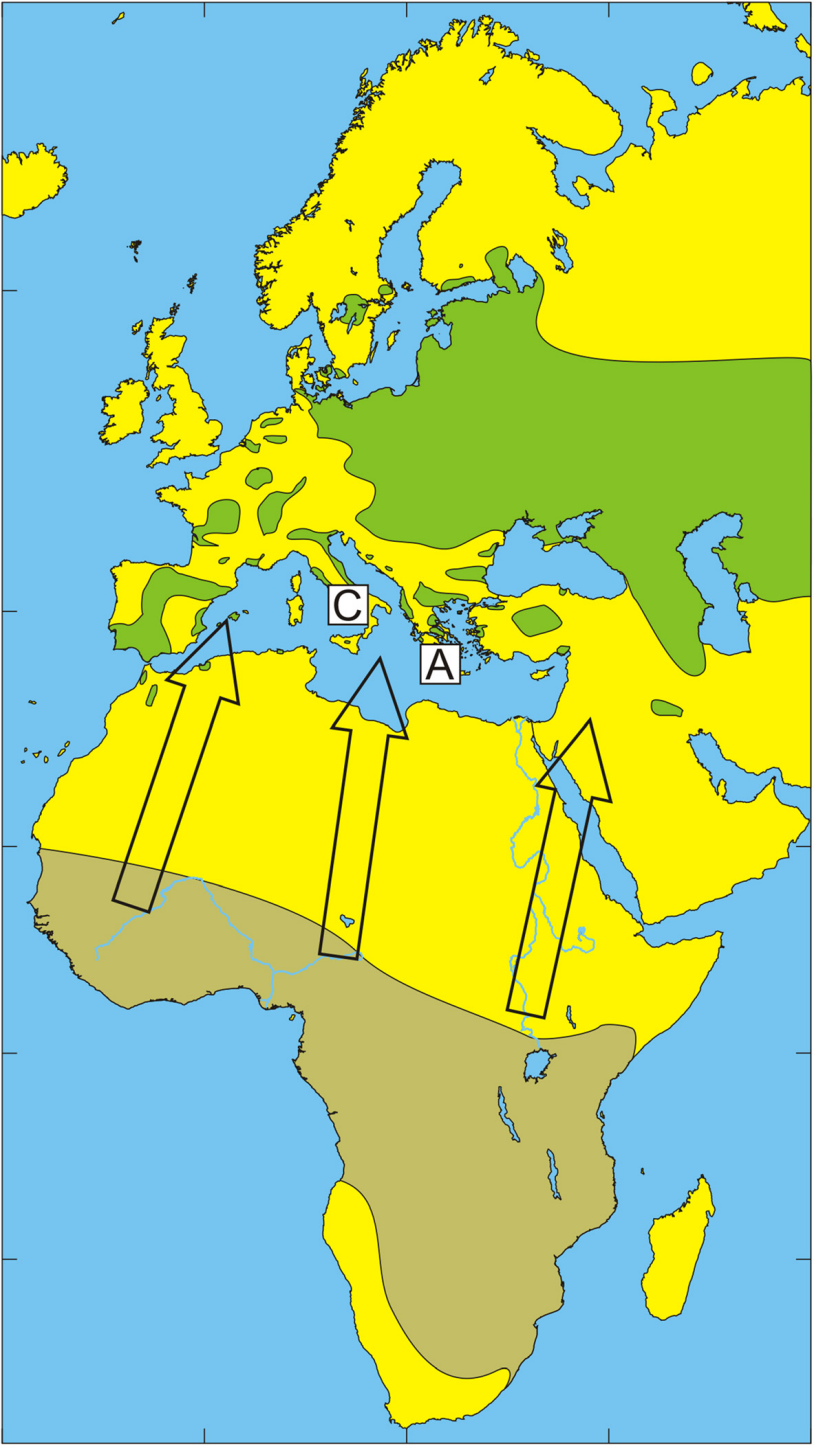
**Illustration of the wintering and breeding areas and migration directions of five bird species.** Breeding (green) and wintering (light brown) areas for some bird species included in the study, based on Cramp and Perrins [36-38]. Arrows show the main direction of movements from wintering areas towards breeding areas during spring migration. Great Reed Warbler *A. arundinaceus*. The locations of Capri (C) and Antikythira (A) bird observatories in Italy and Greece, respectively, are shown by white squares.

**Figure 5:**
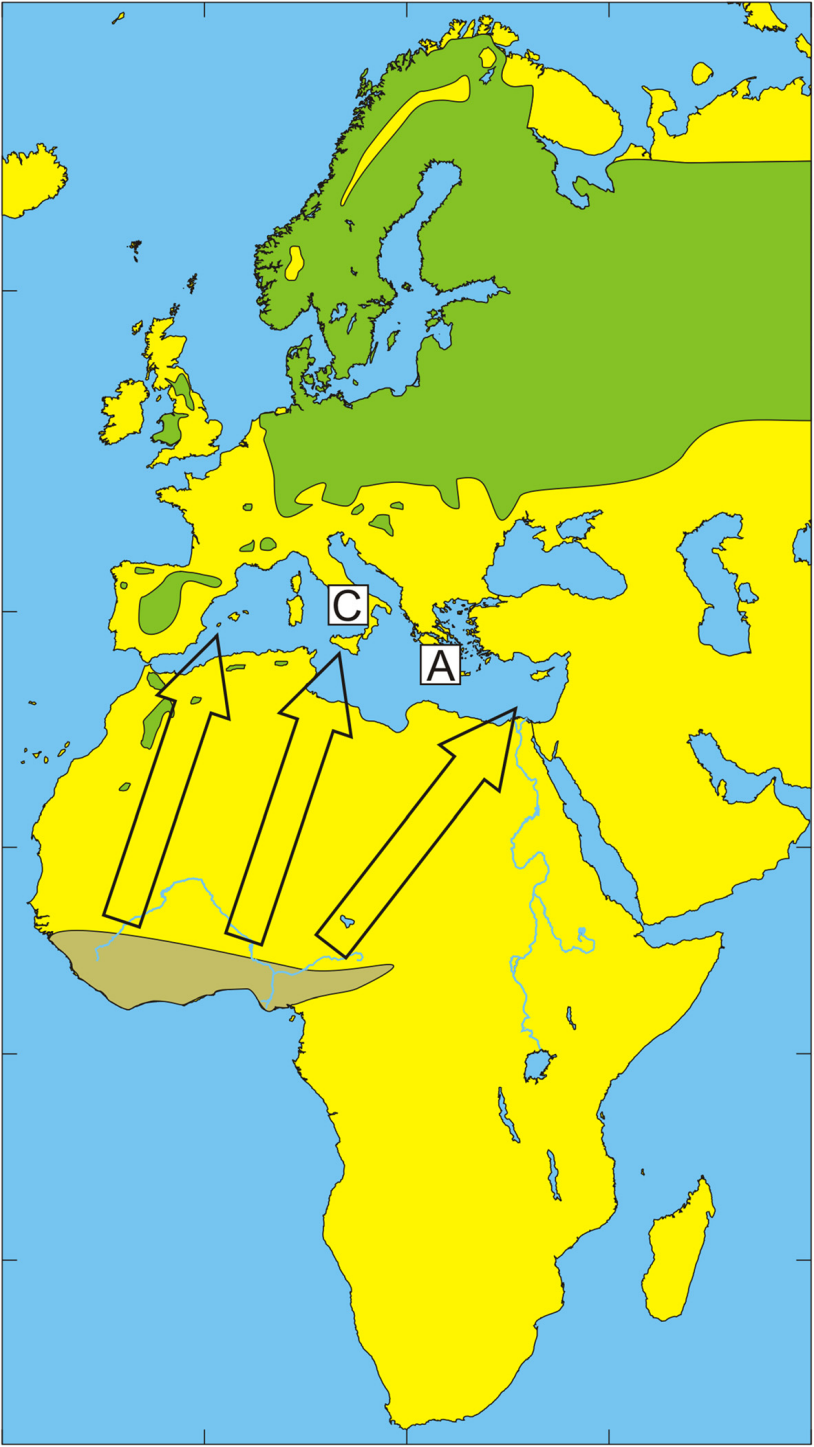
**Illustration of the wintering and breeding areas and migration directions of five bird species.** Breeding (green) and wintering (light brown) areas for some bird species included in the study, based on Cramp and Perrins [36-38]. Arrows show the main direction of movements from wintering areas towards breeding areas during spring migration. Pied Flycatcher *Ficedula hypoleuca*. The locations of Capri (C) and Antikythira (A) bird observatories in Italy and Greece, respectively, are shown by white squares.

**Figure 6:**
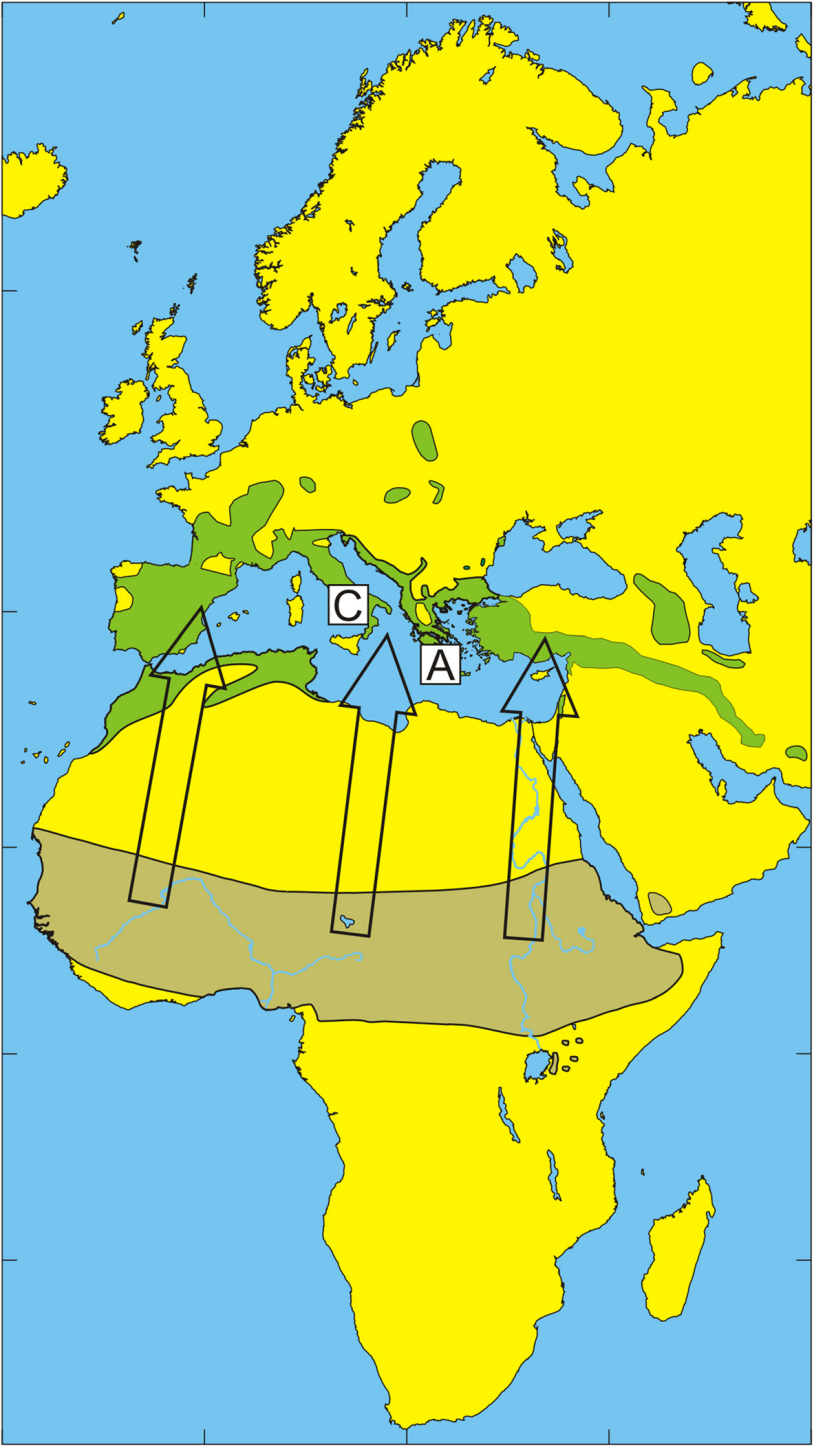
**Illustration of the wintering and breeding areas and migration directions of five bird species.** Breeding (green) and wintering (light brown) areas for some bird species included in the study, based on Cramp and Perrins [36-38]. Arrows show the main direction of movements from wintering areas towards breeding areas during spring migration. Woodchat Shrike *Lanius senator*. The locations of Capri (C) and Antikythira (A) bird observatories in Italy and Greece, respectively, are shown by white squares.

The European bird population includes a few billion birds that migrate annually during spring to their breeding grounds in Europe and return during autumn to their non-breeding grounds in Africa [32]. Species that spend most of their time on the ground in search of food usually have relatively high tick infestation rates [33]. The present study reflects how tick-infested birds may be an important factor for tick dispersal and enhance tick dispersal by carrying infected ticks over long distances to new locations. The geographic distribution of the dominant tick species of the *H. marginatum* complex and migratory routes of observed bird species are summarized in Figures 1, 2, 3, 4, 5 and 6 respectively.

Hard ticks are the primary vectors of several bacterial pathogens, including several species of *Rickettsia*. In the present study the majority of the ticks belonged to the *H. marginatum* complex (=*H. marginatum* s.l*.).* Sequencing results of ten of the ticks suggest that the majority of the ticks belong to the species *H. rufipes*[23]. *H. marginatum* s.l. represented 90% of the total number of ticks; about half of the ticks of this species complex were nymphs. About 4% of the tick collection consisted of other ixodid taxa such as *Ixodes*, *Amblyomma*, *Haemaphysalis* sp. and *Rhipicephalus* sp., the rest were unidentified.

Just about half of all the ticks (48%) were infected with *Rickettsia* spp., with a similar distribution of infected and non-infected ticks among the tick stages. Among the infected ticks, 96% carried *R. aeschlimannii*. Since the *ompB* sequences for *R. aeschlimannii* are identical both years, but different from other annotated sequences, this could indicate that the ticks originated from the same geographic region, possibly because the birds use the same migratory route year after year. Both *R. africae* and *R. aeschlimannii* have been reported from the same African countries [39]. The fact that *R. africae* is the most common and the predominant rickettsial species on the African continent, especially in Sub-Saharan Africa, indicates that the birds carrying the ticks collected in the present study have wintered in northern Africa where *R. aeschlimannii* is more prevalent than in the southern areas. However, *R. aeschlimannii* is endemic in the Mediterranean part of Europe. This rickettsia has been recorded in *H. marginatum* sub-adults and was recently also reported in this tick species removed from a Reed Warbler (*Acrocephalus scirpaceus*) in Germany [12,40]. The main breeding habitats of the Reed Warbler are wetlands and reed beds in Central and Northern Europe from April to October. It winters in Sub-Saharan Africa - including areas inhabited by *H. marginatum* s.l. [41]. Both of these findings support the hypothesis that *Rickettsia* species are imported to new locations by infested birds.

So what are possible consequences of this dissemination of *Rickettsia* species by ornithophagous, usually subadult ticks into Europe? Direct transmission of an infectious agent from wild birds to humans has rarely been recorded [41]. However, immense numbers of ticks are introduced every year to European regions by birds. Successful colonization of alien tick species infected with tick-borne pathogens will depend on suitable environmental conditions for moulting, host-finding, feeding, mating and reproduction. But it still remains to be proven if this way of dispersal occurs. So far, only few human cases of spotted fever caused by *R. aeschlimannii* have been reported from southern Europe [14] and the situation seems to be similar in Africa [42].

*R. africae* is known to be transmitted mainly by different species of *Amblyomma* ticks [43]. However, in the present study all five *R. africae*-infected ticks belonged to *H. marginatum* s.l., *R. africae* has previously been detected in *H. marginatum* collected from cattle in Sicily. This finding of *R. africae* was neither an imported human infection nor an infection in a tick parasitizing birds. In contrast it was interpreted as indicating that *R. africae* is naturally present in Europe [44]. This shows the complexity of the subject and that there is more to explore.

Some species of *Ixodes* ticks were collected in this study, the majority (23/27) were *I. frontalis.* Six of these *Ixodes* specimens were infected with *Rickettsia* spp., and one of these ticks was collected from a Pied Flycatcher, a migratory bird that breeds in much of Europe and western Asia and during winter quarters in tropical Africa. The tick carried a *Rickettsia* sp., possibly *R. monacensis*, previously reported from *I. ricinus* in Germany and other countries in southern Europe as well as from ticks in Korea. *R. monacesis* has been associated with a Mediterranean spotted-fever like illness in humans [45-47]. The other four infected *I. frontalis* ticks harboured *R. aeschlimannii* and infested three birds, two of the ticks infested a Golden Oriole, one came from a Tree-Pipit and one from a Wood Warbler. All these bird species spend the winter in Africa.

Regarding the infestation of individual bird species, as an example, one hundred and thirty-three individuals of the European Robin (*Erithacus rubecula*) were caught in the nets in 2009 and 2010. Eight birds (6.0%) were infested by ticks and three of these birds also carried *I. frontalis*. None of these ticks were infected by *Rickettsia*. This is to be compared with a previous study from Ottenby, Sweden, where 15% of the 418 Robins carried infected ticks (*I. ricinus*), of which 6.9% were infected with *R. helvetica*[4]. The European Robin occurs in Eurasia and has probably had a similar distribution during the past two centuries. The southern border of the breeding area is in North Africa and the Azores and Madeira, which also forms the western border, while the Scandinavian and Russian robins spend the winter in the UK and Western Europe.

Some of the birds were infested with more than one tick, usually fully fed ticks, and the vast majority was infected with the same rickettsial species. For example, a Woodchat Shrike, which usually winters in Central Africa, south of the Sahara and north of the Equator, had 19 engorged ticks, four of which were negative for rickettsial DNA while 15 were infected with *R. aeschlimannii*. This may indicate that their avian host was rickettsiaemic and that the ticks had been infected through their blood meals. Another example was a Common Whitethroat infested by four engorged *Hyalomma* larvae, all infected with *R. aeschlimannii.* On the other hand, some birds carried several ticks that were infected with different *Rickettsia* species: for example one Sedge Warbler caught on Antikythira in 2009 was infested by eight ticks, three were negative, another three were infected with *R. aeschlimannii* and two ticks shared the same identical undefined *Rickettsia* sp. Some birds carried more than one tick of which only one individual tick was infected with a *Rickettsia* species. These findings do not support the view that birds may serve as rickettsial reservoirs, but it is likely that they sometimes have a rickettsiaemia that contributes to the transmission of the bacteria among feeding ticks [4]. This assumption is supported by a recently reported study that shows that birds may be bacteriaemic with *R. helvetica* and act as a reservoir [48].

In two ticks sequences of rickettsial origin were found, however, the lengths of the fragments were too short to enable species differentiation. For the remaining five ticks positive for *Rickettsia* but different from *R. aeschlimannii* and *R. africae* the sequence results were too inconclusive to determine the *Rickettsia* species with certainty. It is possible that these ticks had multiple infections with different *Rickettsia* spp. or that some of them had a *Rickettsia* sp. that has not been completely defined yet or they might even have had new undefined *Rickettsia* species.

The number of ticks brought by different bird species from Africa to Europe during spring migration varies. This study contains a large number of birds, and the frequency at which they were caught can be assumed to show some coherence with the total composition of spring migrating birds in the area. Some bird species were caught in such low numbers that it is hard to hypothesise how much they contribute to the dispersal of ticks in Europe. But there are some trends that can be highlighted. A total of 122 Woodchat Shrikes brought with them 53 ticks, which makes it the most heavily infested bird species in this collection; one individual bird was infested by 19 ticks alone. Though this species is not among the most common ones the total amount of ticks is high in relation to other species. The Woodchat Shrike was mostly caught at Antikythira. For the Common Redstart there is a similar importance with 51 ticks carried by 386 individual birds. Both species spend much time on the ground and each individual bird has a high risk of becoming tick-infested during its seasonal migration. This can be compared with the Blackcap (*Sylvia atricapilla*) with 248 individuals caught and no tick found (not included in Table 2).

Six species of birds where caught in more than 1,000 individuals per species and can be considered common (Table 2). There is a difference in how great their tick infestations were; the Garden Warbler is the most commonly caught bird in this project with 2,190 individuals and which were only infested by a total of 13 ticks. This is comparable with the Spotted Flycatcher (*Muscicapa striata*) of which 1,187 individual birds carried a total of only 7 ticks. In contrast, the Whinchat that spends much of its time in close proximity to the ground, contributed to the highest number of ticks imported by a single bird species, i.e., by 135 ticks on 1,472 birds. The same goes for the Common Whitethroat, which brings in approximately the same number of ticks per bird (123 ticks on 1,244 birds) and just slightly less common as the Whinchat in this material. In total the Whinchat and the Common Whitethroat contributes to the largest burden if we assume that the relation between captured birds in this project reflects the proportions between all migrating birds in the area.

## Conclusions

Migratory birds appear to have a substantial impact on the spread and local presence of ticks infected with *Rickettsia* species, some of which may originate from distant locations. The potential ecological, medical and veterinary implications of the dissemination of such infections and the role of birds in the epizootiology and epidemiology of tick-borne pathogens in this area needs to be further examined.

## Abbreviations

bp: Base pair; nt: Nucleotide; s.l.: Sensu lato.

## Competing interests

The authors declare that they have no competing interests.

## Authors’ contributions

Conceived and designed the study: KW, BO, CB, ES, KN. Performed the experiments: KW, FN, PEL. Identified the ticks: TGTJ. Analysed the data: KW, BO, TF, TGTJ, KN. Contributed reagents/materials/analysis tools: BO, PEL, KN. Wrote the paper: KW, TGTJ, KN. All co-authors co-revised the manuscript, co-refined the intellectual content of the manuscript and approved the final version.
